# NIR multiphoton ablation of cancer cells, fluorescence quenching and cellular uptake of dansyl-glutathione-coated gold nanoparticles

**DOI:** 10.1038/s41598-020-68397-1

**Published:** 2020-07-09

**Authors:** Antonio Buonerba, Rosita Lapenta, Anna Donniacuo, Magda Licasale, Elena Vezzoli, Stefano Milione, Carmine Capacchione, Mario Felice Tecce, Andrea Falqui, Roberto Piacentini, Claudio Grassi, Alfonso Grassi

**Affiliations:** 1 Sanitary Environmental Engineering Division (SEED), and Consorzio Inter-universitario Previsione e Prevenzione dei Grandi Rischi (Cu.G.Ri.), Department of Civil Engineering, University of Salerno, Via Giovanni Paolo II, 84084 Fisciano, SA Italy; 2 Department of Chemistry and Biology “Adolfo Zambelli”, University of Salerno, Via Giovanni Paolo II, 84084 Fisciano, SA Italy; 3 Biological and Environmental Sciences and Engineering (BESE) Division, King Abdullah University of Science and Technology (KAUST), Thuwal, 23955-6900 Saudi Arabia; 4 Department of Pharmacy, University of Salerno, Via Giovanni Paolo II, 84084 Fisciano, SA Italy; 5 Department of Neuroscience, Università Cattolica del Sacro Cuore, Rome, Italy; 6 Fondazione Policlinico Universitario A. Gemelli IRCCS, Rome, Italy

**Keywords:** Cancer therapy, Cancer, Nanotechnology in cancer, Other photonics

## Abstract

Theranostics based on two-photon excitation of therapeutics in the NIR region is an emerging and powerful tool in cancer therapy since this radiation deeply penetrates healthy biological tissues and produces selective cell death. Aggregates of gold nanoparticles coated with glutathione corona functionalized with the dansyl chromophore (a-DG-AuNPs) were synthesized and found efficient nanodevice for applications in photothermal therapy (PTT). Actually the nanoparticle aggregation enhances the quenching of radiative excitation and the consequent conversion into heat. The a-DG-AuNPs are readily internalized in Hep G2 where the chromophore acts as both antenna and transducer of the NIR radiation under two-photons excitation, determining efficient cell ablation via photothermal effect.

## Introduction

Theranostics allowed a transition from conventional to personalized and precision medicine in cancer therapy. The cell monitoring can be performed at low drug dosage along with the selective cell ablation, reducing side effects and risks for the oncological patients during therapy^1,2^. One of the emerging approach is the plasmonic photothermal therapy (PTT) consisting in nanoparticle-assisted hyperthermia under laser irradiation of appropriate wavelength^3^. The surface plasmon resonance (SPR) of noble metal nanoparticles produces hot electrons on the nanoparticle surface under visible or near-infrared (NIR) excitation; the heat generated through photon-photon interactions is rapidly transferred from the surface to the environment, determining a temperature increase of tens of degrees in the cell compartment where the nanoparticles are located. Compared to more energetic radiations (e.g. UV light) the NIR light offers several advantages, such as minimal invasiveness, rapid recovery, fewer complications and much deeper penetration in tissues^4^. PPT has been successfully performed in in vivo and in vitro experiments using gold-based nanomaterials^5–12^, e.g. star or rod-shaped nanoparticles, nanocages and nanoshells that show intense absorption in the NIR region; however, these nanodevices need sophisticated synthetic procedures and are typically prepared in small scale^13,14^. Spherical AuNPs are simple in structure, commercially available or synthesized with a variety of capping agents and functionalized shell showing low toxicity^15–18^. An intense plasmonic absorption is observed typically at about 520 nm; direct CW excitation to this wavelength (10–15 min) is converted into heat leading to irreversible cell damage as a result of thermal denaturation of proteins, mechanical stress of the membrane or bubble formation^19^. This absorption dramatically decreases in the range of 800–1,000 nm where poor performances in PPT were observed under laser excitation^5–10,13,14^.

Glutathione coated spherical AuNPs (G-AuNPs) have been extensively investigated as drug delivery system because of their low affinity to serum proteins^20^: they are photoluminescent and exhibit size independent emission at 600 nm and 800 nm when excited at 396 nm and 350 nm, respectively. Moreover a strong electrostatic adhesion to the cell membrane was observed in Hep-G2^21,22^.

In this paper, we show that both G-AuNPs and AuNPs coated with glutathione functionalized with dansyl fluorophore (DG-AuNPs) are readily internalized, even into the nucleus, in human hepatocytes Hep G2 cells and murine neuronal cells as aggregates of well-defined size. After AuNPs internalization, irradiation with pulsed-laser in the NIR spectral window induced efficient cell ablation that was greater with DG-AuNPs than with G-AuNPs.

## Results

**Figure 1:**
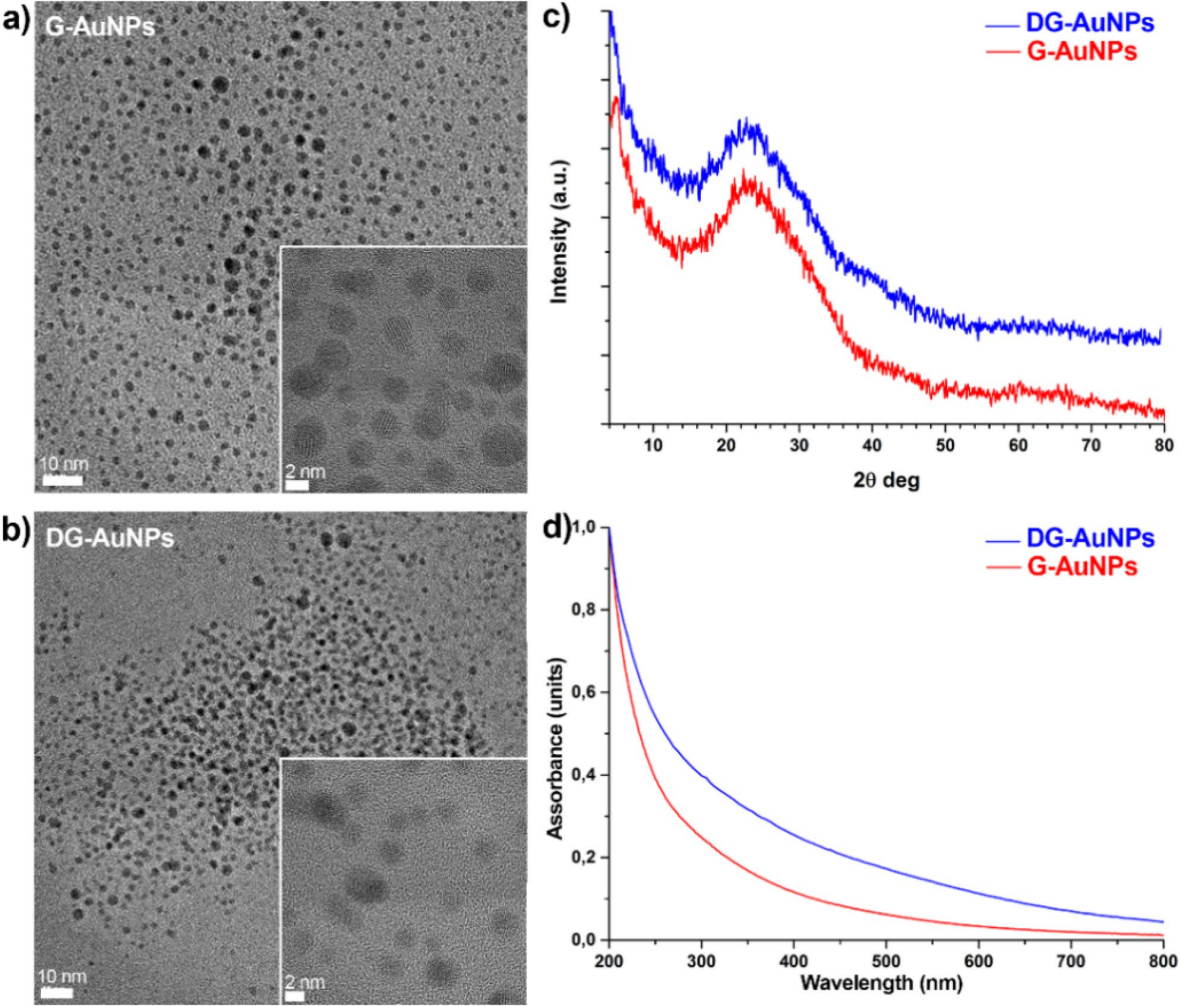
TEM micrographs of G-AuNPs (**a**) and DG-AuNPs (**b**). Powder WAXD diffractograms (**c**) and UV–Vis-NIR spectra (**d**) of G-AuNPs and DG-AuNPs.

The G-AuNPs and DG-AuNPs were synthesized according to the literature procedure^23,24^ and recovered from the hydroalcoholic solution using a salt-induced precipitation with sodium chloride and methanol. The transmission electron microscopy (TEM) analysis shows that the synthesized nanoparticles are of mean size of 2.5 nm with narrow size distribution (Fig. 1a,b); in agreement with this result, the reflections for crystalline (face-centred cubic, *fcc*) gold (Fig. 1c) expected in the powder wide-angle x-ray diffraction (WAXD) pattern (Fig. 1c)^25^, and the typical plasmonic band at ≈ 520 nm, in the UV–Vis spectra (Fig. 1d), are too broad to be detected^26^.

**Figure 2:**
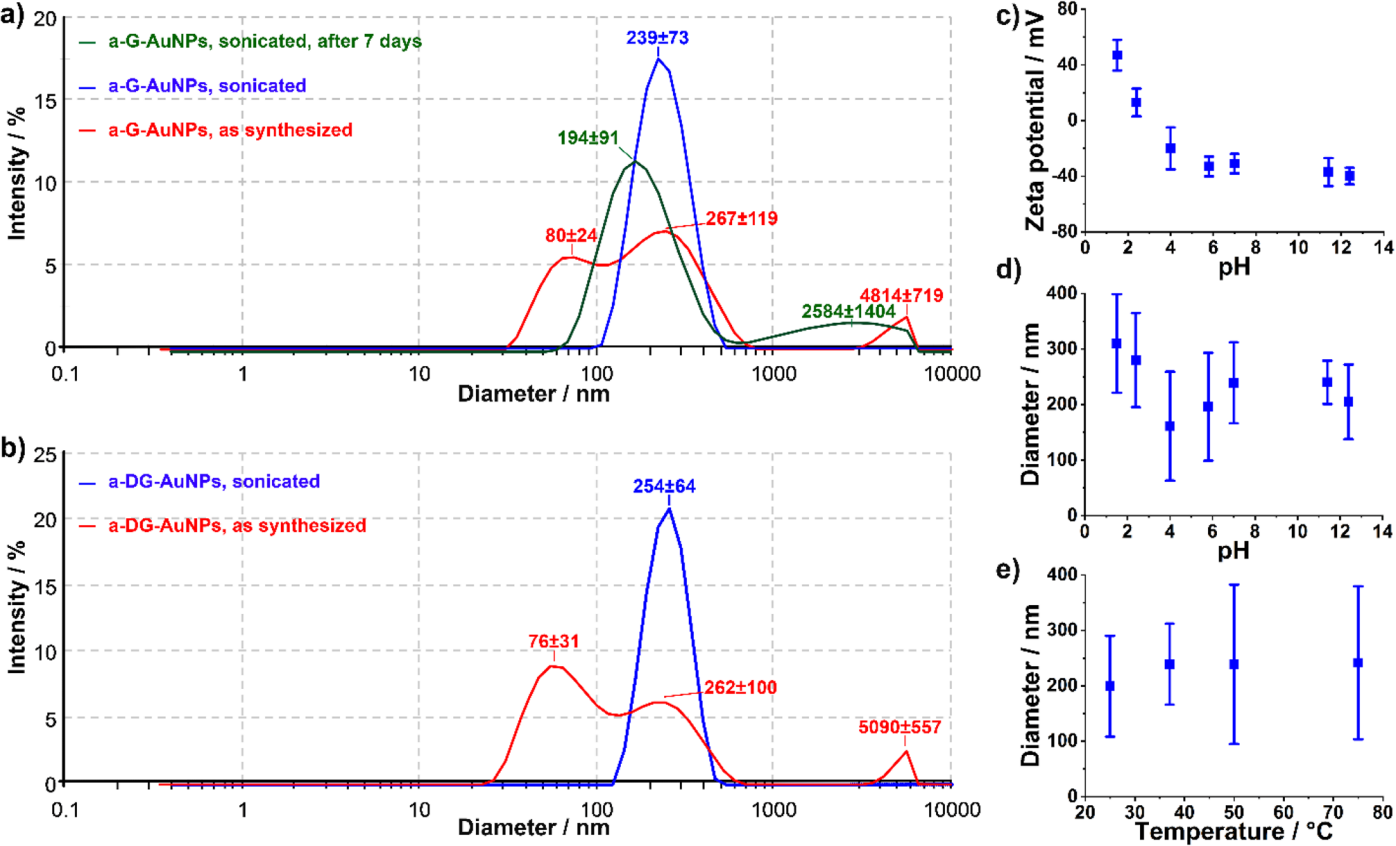
DLS measurements. Size distribution profiles of G-AuNPs (**a**) and DG-AuNPs (**b)**. Zeta potential (**c**) and diameter (**d**) of G-AuNPs as a function of pH. Diameter (**e**) of G-AuNPs as a function of temperature.

Hydrodynamic size and size distribution were assessed in deionized water or phosphate-buffered saline (PBS) solutions by dynamic light scattering (DLS) using a concentration of 700 µg/mL. In both the cases, the size distribution profiles suggest the formation of nanoparticle aggregates with different hydrodynamic size (Fig. 2a,b). However, mono-modal curves centred at 239 ± 73 nm and 254 ± 64 nm were observed for the G-AuNPs and DG-AuNPs aggregates (a-G-AuNPs and a-DG-AuNPs; Fig. 2a,b) after sonication for 30 min at 45 kHz. The zeta potentials of − 32 ± 5 mV and − 29 ± 5 mV (Fig. 2c), suggest negatively charged surface and good colloidal stability; in fact, the aggregates are dimensionally stable for at least one week (Fig. 1a), independently of the capping agent, composition and experimental conditions. Considering that the pH values range from slightly basic in healthy biological cells to acidic in cancer cells^27^, the stability of the aggregates was screened in buffer solutions in a wide range of pH 1.5–12.4 (Fig. 2d). At the isoelectric pH of glutathione (pI = 5.93) a-G-AuNPs show a size of 196 ± 97 nm and zeta potential of − 33 mV (Fig. 2c) whereas at pH of 2–3 the zeta potential becomes neutral causing an increase of the size (compare Fig. 2c,d). In all investigated cases, the size of the nanoparticle aggregates is independent of temperature in the range 25–75 °C (Fig. 2e).

**Figure 3:**
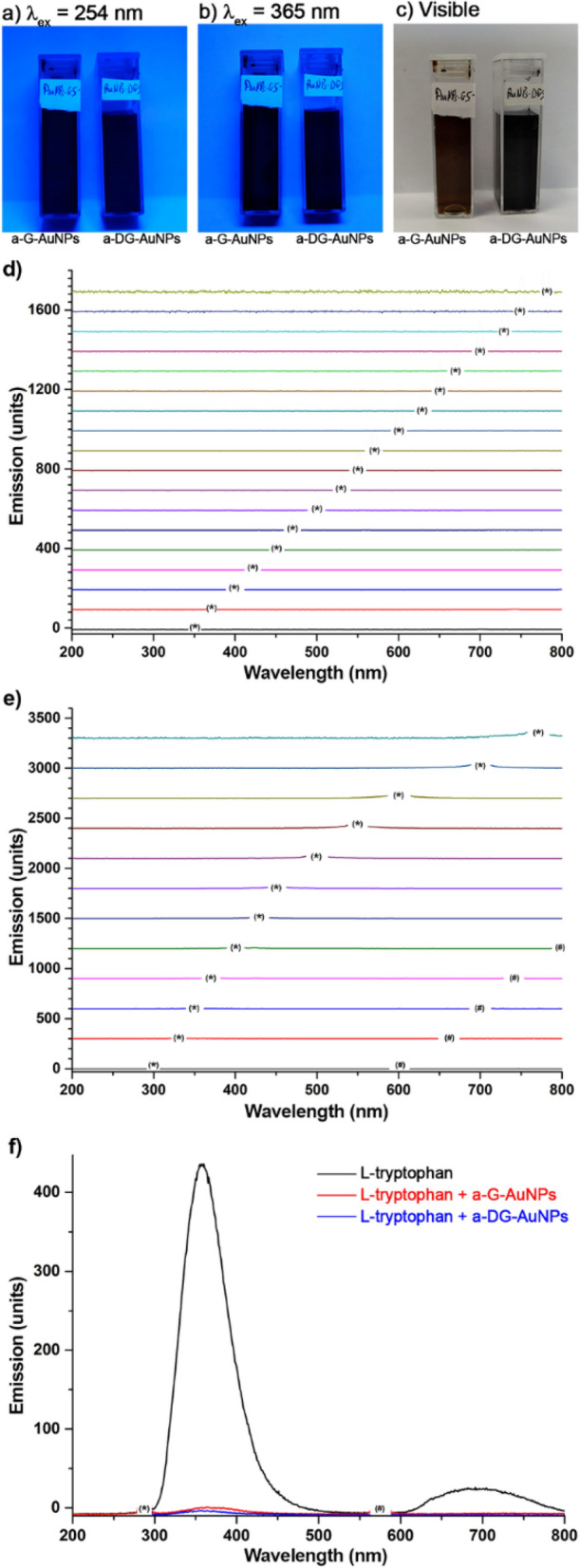
Aqueous suspensions of a-G-AuNPs and a-DG-AuNPs (700 μg/mL) under UV (**a**,**b**) and visible light (**c**) irradiation. Emission spectra of a-G-AuNPs (**d**) and a-DG-AuNPs (**e**) at variable wavelengths of excitation. Emission spectra with excitation at 287 nm (**f**) of l-tryptophan (1.5 × 10^–5^ mol/L) in absence and in presence of a-G-AuNPs or a-DG-AuNPs (125 µg/mL). * λ_ex_; ^#^ Diffraction at 2λ_ex_ due to diffraction from the grating of the spectrometer monochromator.

Under UV–vis excitation, G-AuNPs and DG-AuNPs do not show any emission band (see Fig. 3) both in the solid state and colloidal suspension. When an external chromophore, e.g. l-tryptophan (6.5 mg/mL, 0.03 mol/L), was added to the water suspensions of AuNPs (175 µg/mL), a complete quenching of the strong fluorescence of the amino acid was observed (Fig. 3f). The Stern–Volmer plot of fluorescence quenching of tryptophan (1.5 × 10^–4^ M) at variance of a-G-AuNPs concentration at 22 and 70 °C indicates a dynamic quenching of fluorescence (see Figure S5). Increasing the temperature from 22 to 70 °C, the Stern–Volmer constant (*K*_*D*_) changed from 0.179 ± 0.006 L/mg_AuNPs_; to 0.195 ± 0.008 L/mg_AuNPs_. Higher temperatures resulted in faster diffusion of fluorophore and AuNP quencher and hence in larger amounts of collisional quenching^28^.

In vivo toxicity, biodistribution and clearance of glutathione-coated gold nanoparticles have been extensively investigated^24^. The toxicity of a-G-AuNPs and a-DG-AuNPs was screened using human liver cancer cell, Hep G2 line, treated with aqueous suspensions of the aggregates at the concentration of 800 µg/mL for 1–24 h. After a small initial flexion (1 h), observed also in the control experiment, the cell viability resulted unaffected by the treatment (see Figure S1). The gold uptake was investigated by inductively coupled plasma-optical emission spectroscopy (ICP-OES) analysis of acidic digested Hep G2 cells exposed for 1 h to a-G-AuNPs (2.0 mL, 80 µg/mL) and rinsed from the culture medium containing the excess of AuNPs (see experimental section for details); a concentration of 2.6 ± 1.2 pg/cell corresponding to ≈ 8.0 aggregates/cell was measured assuming the size determined by DLS measurements and the glutathione/gold molar ratio of 0.4–0.6 assessed by thermogravimetric curves (Figures S2–S4) and ICP-OES analyses.

**Figure 4:**
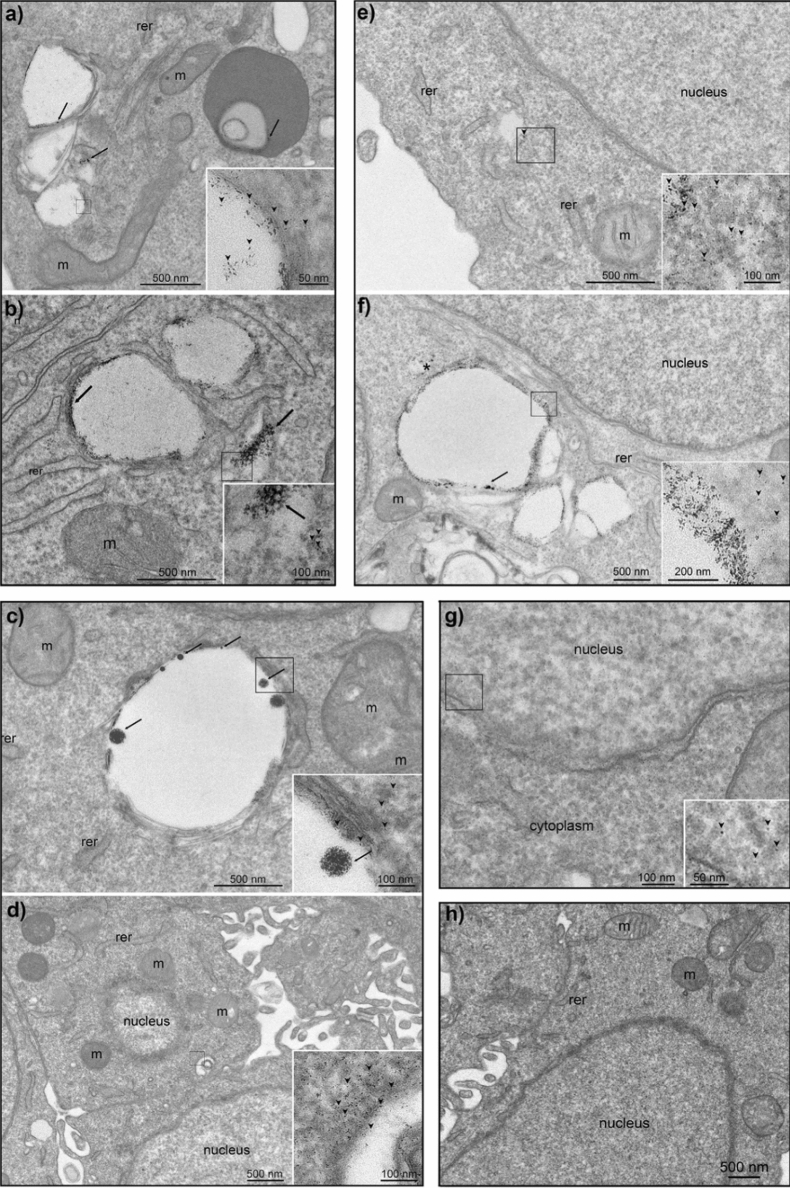
TEM micrographs of Hep G2 cells treated with aqueous suspensions of: a-G-AuNPs with the concentration of 70 µg/mL for 1 h (**a**) or 2 h (**b**); a-DG-AuNPs with the concentration of 70 µg/mL for 1 h (**c**) or 2 h (**d**); a-G-AuNPs (**e**) or a-DG-AuNPs (**f**) both with the concentration of 700 µg/mL for 1 h. Magnification of Hep G2 cells treated with a-G-AuNPs (70 µg/mL; for 1 h) evidencing the internalization of the AuNPs into the nucleus [black arrowheads in inset of (**g**)]. Control experiment of Hep G2 cells not exposed to the nanoparticles (**h**). (m = mitochondria; rer = rough endoplasmic reticulum, n = nucleus).

Cell cultures of Hep G2 were seeded into plates and treated for 1 h and 2 h with 1.0 mL of 70 µg/mL or 700 µg/mL suspensions of a-G-AuNPs or a-DG-AuNPs, at a cellular confluence of 80–90%. The TEM image of the cells exposed for 1 h to a-G-AuNPs (70 µg/mL) shows aggregates of ~ 50 nm (black arrows in Fig. 4a) internalized in vesicles as well as several single nanoparticles located in the cytoplasm, organelles and nucleus (black arrowheads in Fig. 4a,g). After an incubation time of 2 h, the aggregates were detected in some vesicular structures and in the cytosol (black arrows in Fig. 4b) along with single nanoparticles (black arrowheads in inset of Fig. 4b). Cells exposed for 1 h to a-DG-AuNPs (70 µg/mL) contain large aggregates of 50–100 nm inside vesicles (black arrows in Fig. 4c), while several single nanoparticles are located in the cytoplasm (black arrowheads Fig. 1e and its inset). After a prolonged incubation of 2 h, a higher number of isolated AuNPs are detected in the cytoplasm (black arrowheads) (Fig. 4d). At higher concentration (700 µg/mL), a number of vesicles containing aggregates and isolated AuNPs are found in the cytoplasm (Fig. 4e,f). The internalization of large nanoparticle aggregates was also observed at such a concentration of a-DG-AuNPs (Fig. 4f). These findings suggest that the internalization process occurs via invagination of vesicles containing the aggregates and this process does not affect the cell membrane integrity. Noteworthy, when exposed to the intracellular environment, the aggregates release isolated AuNPs that are able to penetrate the nucleus (see Fig. 4g).

**Figure 5:**
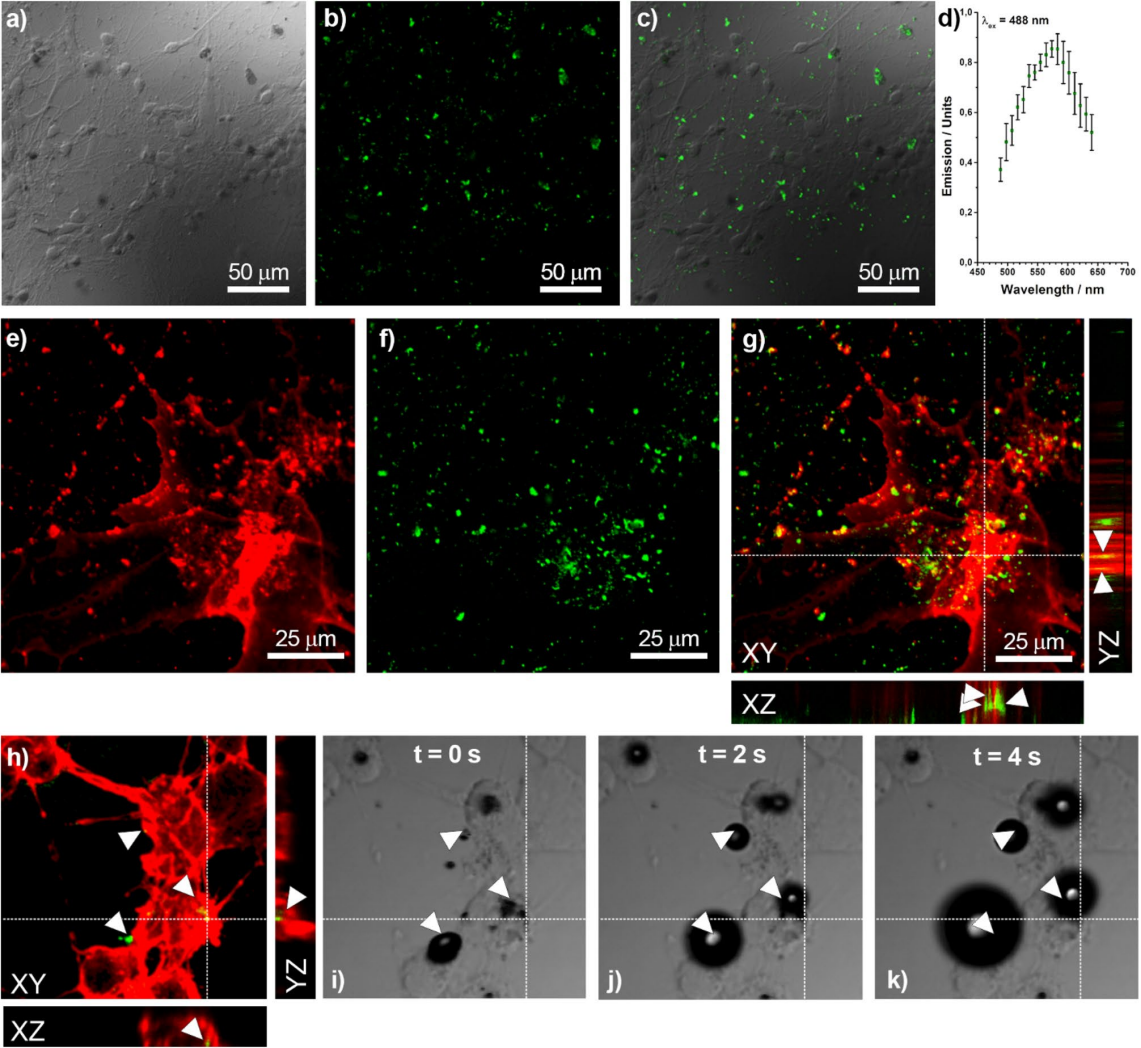
Imaging at confocal microscope of primary cultures of murine cortical neurons and astrocytes incubated with a-DG-AuNPs (700 µg/mL) for 4 h. *Identification of DG-AuNPs and fluorescence*: (**a**) DIC image (×40); (**b**) fluorescence image of AuNPs (λ_ex_ = 488 nm); (**c**) merging of the images **a** and **b** showing the correspondence between the nanoparticle location and green fluorescence; (**d**) emission spectrum of AuNPs (λ_ex_ = 488 nm). *Internalization in cell of DG-AuNPs*: (**e**) fluorescence image of astrocytes with rhodamine-stained F-actin (red); (**f**) fluorescence image of AuNPs (λ_ex_ = 488 nm); (**g**) merging of images **d** and **e**, with Z-stacking lateral views showing the internalization of the AuNPs in the cell. *Cell photoablation:* (**h**) fluorescence image of neuron with rhodamine stained F-actin; (**i**–**k**) DIC images upon laser-pulsed excitation tuned at wavelength of 760 nm and power of 24 W/cm^2^ showing the rapid formation of bubbles in correspondence of a-DG-AuNPs.

The internalization of G-AuNPs and DG-AuNPs in Hep G2 cells was also investigated by high-resolution confocal microscopy; for seek of generality, the same study was extended to murine cortical neurons and astrocytes, which show higher negatively charged membrane (− 33 mV in Hep G2 vs. − 80 mV in neurons and − 71 mV in astrocytes) to demonstrate that the electrostatic repulsion of the membrane is overcome by the nanoparticle aggregates. Primary cultures of murine cortical neurons and astrocytes were treated for 4 h with a-G-AuNPs and a-DG-AuNPs (0.5 mL; 700 µg/mL), fixed with para-formaldehyde, washed with PBS and imaged, after inclusion in an antifade mounting medium. Large aggregates of AuNPs were identified as dark spot in the differential interference contrast (DIC) images (Fig. 5a) and found emitting fluorescence in the spectral range 500–650 nm (Fig. 5b–d) after excitation at 488 nm with continuous wave (CW) laser light (Fig. 5d). An intense fluorescence was observed for DG-AuNPs with respect to G-AuNPs (compare Figures S5a and S6a in the Supporting Information). Figures 5e–g compare the fluorescence of rhodamine-conjugated F-actin used for staining the cytoskeleton of the neuronal cell (Fig. 5e) and the fluorescence emitted from DG-AuNPs (Fig. 5f) with the corresponding merging (Fig. 5g). The lateral projections of the cell (XZ and YZ insets in Fig. 5g), generated by stacking of images acquired by moving the confocal plane along the perpendicular Z-axis, show the effective internalization of the AuNPs in the cells.

The threshold response of a-G-AuNPs and a-DG-AuNPs to NIR excitation was investigated with a confocal microscope after incorporation in neuronal cells and after irradiation for 1.26 s with pulsed laser tuned at 760 nm. The formation of micrometric sized bubbles was observed in correspondence of the a-DG-AuNPs at laser power output of 24 W/cm^2^, corresponding to a fluence of 30 J/cm^2^ (see Figs. 5h–k and S6); at the same laser power, a-G-AuNPs are inactive and get started responding only at a fluence of about 220 J/cm^2^ (see Figure S7).

**Figure 6:**
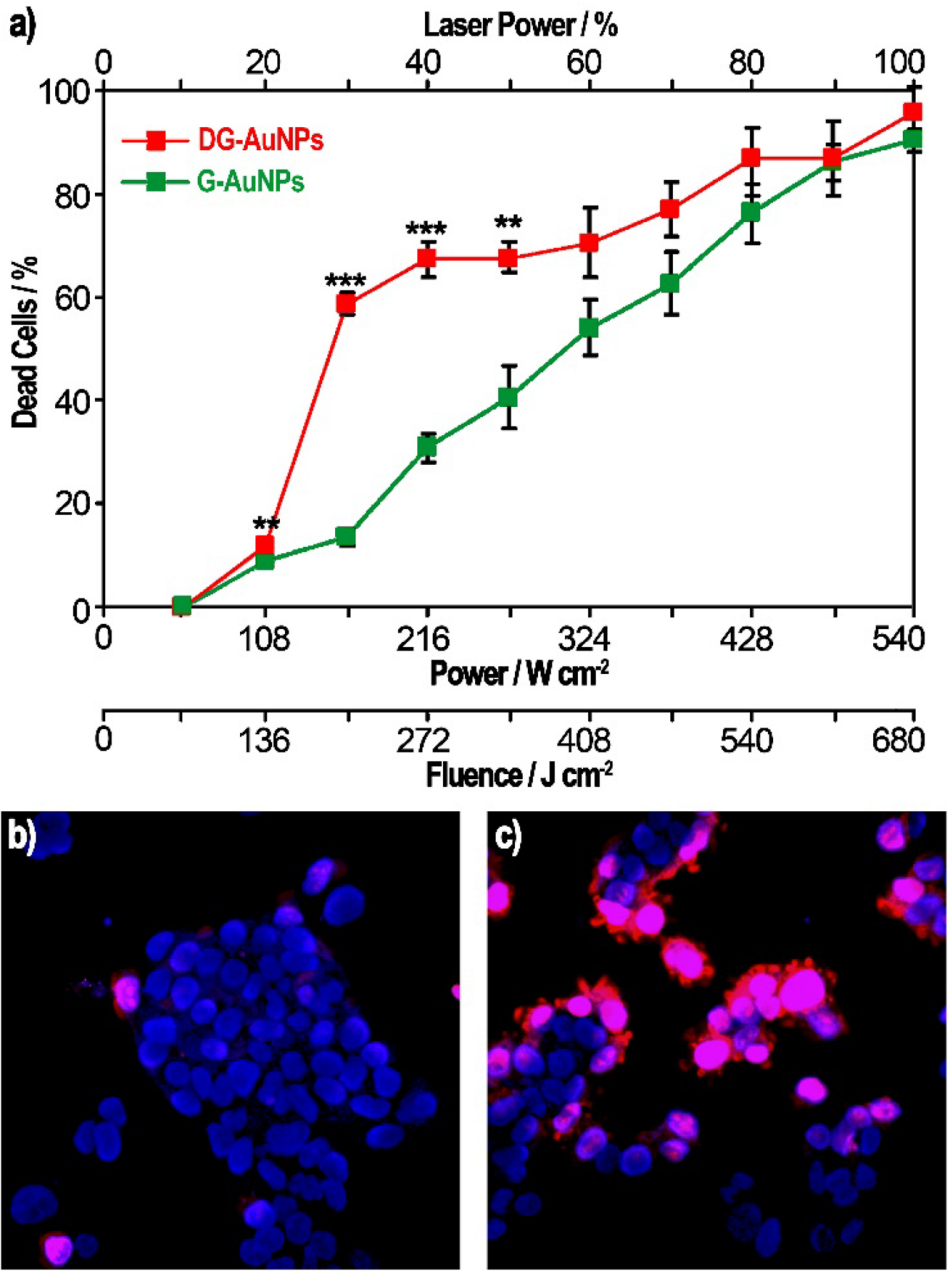
Mortality of Hep G2 cells (**a**) after irradiation for 1.26 s at different incident powers with pulsed laser tuned at 760 nm (determined as percentage of PI-stained cells/total cells; **p < 0.01; ***p < 0.001). Representative images of Hep G2 cells, treated with Hoechst (blue) and propidium iodide (red) stains (colocalization being violet), irradiated for 1.26 s with pulsed laser tuned at wavelength of 760 nm and power of 270 W/cm^2^ (50% laser power) in absence of AuNPs (**b**) or after incubation with DG-AuNPs at concentration of 70 µg/mL for 1 h (**c**).

Cancerous Hep G2 cells were incubated in different in plates for 1 h with a-G-AuNPs or a-DG-AuNPs at the concentration of 70 µg/mL and excited for 1.26 s with a pulsed laser tuned at wavelength of 760 nm and different incident power values; the plot of cellular mortality is shown in Fig. 6a. To differentiate the dead and healthy cells under confocal microscopy (Fig. 6b,c) the plates were treated with propidium iodide (PI) and Hoechst stain. The a-DG-AuNPs were found highly effective in the cell ablation showing 60% of cells positive to the PI test at fluence of ≈ 200 J/cm^2^ (see Fig. 6a); under the same conditions, the mortality of cells treated with a-G-AuNPs was of about 10%. A convergence of the photoablation effects is observed at higher laser fluence, where both the systems result effective in the cell ablation. Vehicle-treated cells, used as control experiments, do not show significant photoablation under similar irradiation conditions (compare Fig. 6b with 6c).

**Figure 7:**
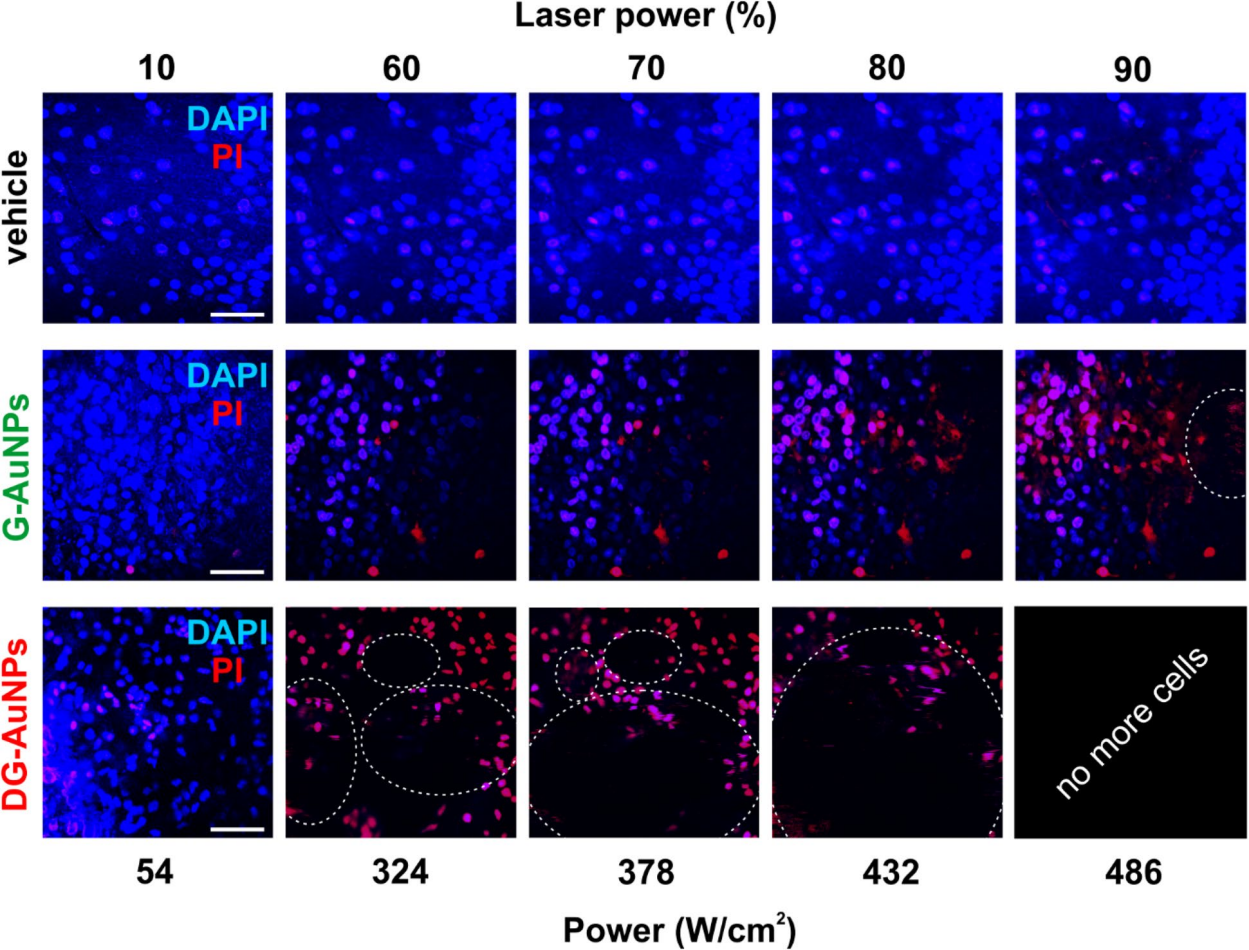
Organotypic brain slices (350-µm thick) treated for 1 h with the vehicles, G-AuNPs and DG-AuNPs (0.7 mg/mL) and irradiated with pulsed laser tuned at 760 nm at various power. DAPI was used to identify cell nuclei, and PI for cell death. Dotted circles represent heat-formed bubbles generated soon after irradiation. Scale bar: 50 µm.

To validate our results, we repeated experiments in a more complex experimental model, such as organotypic brain slices^29^. They were treated with vehicle, a-G-AuNPs or a-DG-AuNPs (700 µg/mL) for 1 h followed by DAPI treatment for the identification of cell nuclei, and PI for evaluating the irradiation-induced cell death. Noteworthy, the brain slices employed in this study retain the anatomical structure of brain for a thickness of 350 µm. As shown in Fig. 7, the photothermal effect was higher in the brain slices treated with DG-AuNPs where bubbles are readily generated after irradiation.

## Discussion

**Figure 8:**
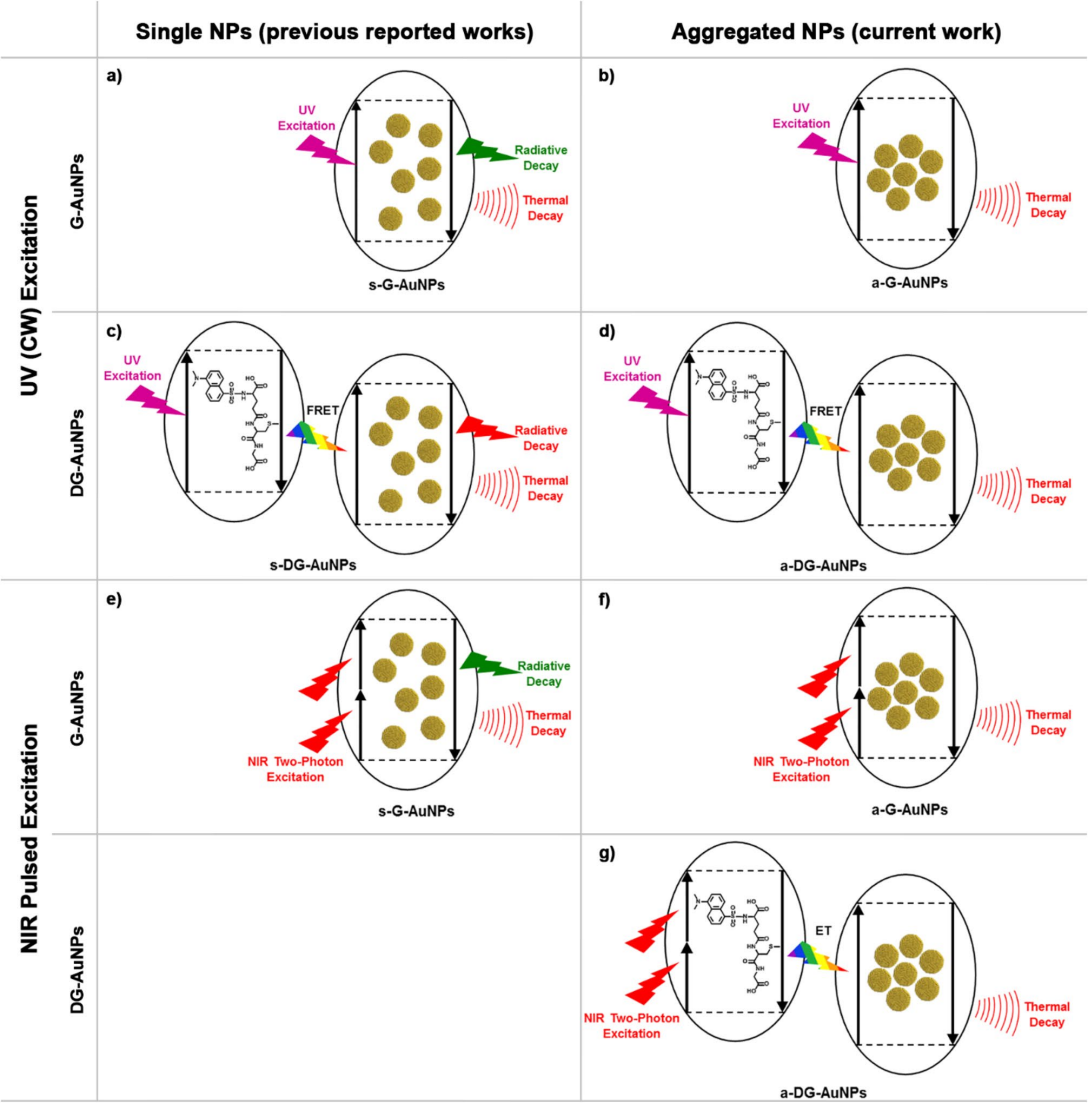
Photophysics of s-G-AuNPs, s-DG-AuNPs, a-G-AuNPs and a-DG-AuNPs under UV (**a**–**d**) and NIR two-photon excitation (**e**–**g**).

The salt-induced precipitation of a-G-AuNPs and a-DG-AuNPs yields the formation of aggregates in which the photophysical properties and the internalization rate in cancer cells are strongly different from those of singularly dispersed spherical nanoparticles^30,31^. Indeed the latter are luminescent upon UV excitation, with size-independent and pH dependent emissions at 600 or 800 nm (compare Fig. 8a with 8b)^21,22^. The strong interparticle plasmonic coupling in the a-G-AuNPs and a-DG-AuNPs shifts the absorption to higher wavelengths^32^ and additionally makes them effective quenchers of the NIR radiation. Due to the broad absorption band in the UV–Vis spectral range, AuNPs are absorbers of dye fluorescence^33^. Singularly dispersed spherical DG-AuNPs show attenuation of the dansyl fluorescence at 400–600 nm, via Förster resonance energy transfer (FRET) with the AuNPs, followed by emission in the range 600–800 nm (see Fig. 8c)^34^, whereas the a-DG-AuNPs show the complete quenching of both dye fluorescence and AuNPs luminescence (see Figs. 3 and 8d).

The a-G-AuNPs and a-DG-AuNPs readily quench the fluorescence of dyes in solution. E.g., l-tryptophan with isoelectric point of 5.65 is electrostatically repulsed at pH values of 7 from the negative charged nanoparticle surface (Fig. 2c) and its fluorescence is totally quenched also when used in large excess. This outcome cannot be attributed to FRET effect but to the energy absorbance of the incoming radiation from the AuNPs aggregates^35,36^. As matter of facts, the Stern–Volmer plot of fluorescence quenching of tryptophan with a-G-AuNPs, ruled out the static quenching of fluorescence, as an evidence that the tryptophan is not adsorbed on the surface of the nanoparticle quencher.

The *“residual”* fluorescence of a-G-AuNPs and a-DG-AuNPs under CW excitation at 488 nm (Fig. 4) in the confocal microscope imaging experiments seems to be in apparent contrast with the efficient fluorescence quenching of aqueous suspensions of AuNPs aggregates. The emission of aqueous solution of single AuNPs is possibly due to the unlikely relaxation via neighbour nanoparticles effect. The fluorescence emission of a-G-AuNPs and a-DG-AuNPs in the cells can be thus tentatively attributed to the surface where the emission is not quenched by local inner filter effect. A similar finding was observed by Pradeep in superlattices of dansyl glutathione protected AuNPs in *fcc* arrangement^37^. Noteworthy the residual fluorescence of a-G-AuNPs and a-DG-AuNPs still allows cell monitoring.

Research efforts have been extensively addressed in the past years toward the search of capping agents which allow fast and controlled nanoparticle aggregation followed by internalization into cells without producing membrane degradation^38^. In this study we report a very simple salt induced aggregation process of a-G-AuNPs and a-DG-AuNPs, and their fast internalization via endocytosis without producing cell death; these outcomes are significantly different from those reported with negatively charged singularly dispersed spherical G-AuNPs which were found exclusively adsorbed onto cell membrane^21^. Interestingly, when internalized in Hep G2, the a-G-AuNPs and a-DG-AuNPs yield in the cytoplasm singular spherical nanoparticles which rapidly penetrate the cell nucleus membrane in short time; further studies are in progress for demonstrating the potential application of such systems in gene therapy.

The AuNPs and the dansyl chromophore can be both excited via NIR multiphoton irradiation^39^. The formation of bubbles in Hep G2 internalized with a-G-AuNPs and a-DG-AuNPs is not accompanied by any radiative emission, as in the case of singularly dispersed G-AuNPs^39^ (see Fig. 8e) or dansylated alkynes^40^. Nanometric bubbles are formed when water is superheated to its spinodal temperature of 277 °C, at which the liquid becomes unstable and spontaneously converts to the vapour phase^41^. Thus, the dansyl fluorophore is excited in a-DG-AuNPs via two-photon upconversion of the NIR radiation; the FRET transfer to the AuNPs aggregates is followed by thermal relaxation (Fig. 8g). Similar effects were observed after irradiation of HOC cells treated with gold nanorods at 800 nm under CW irradiation power of 10 W/cm^2^ for 4 min^4^.

The above-described optical response allows a fictitious widening of the spectral width useful for the photothermal reaction of spherical AuNPs, by enhancing their intrinsically scarce response to NIR excitation. Lanthanide functionalized gold nanoparticles were found similarly effective in cancer therapy using PPT as a result of the same physical effect^42–45^. The NIR two-photon excitation owns significant advantages over direct UV–Vis one-photon excitation of AuNPs because of greater tissue penetration, low autofluorescence levels and low photo damage of healthy tissues^46^.

## Conclusions

The salt-induced precipitation of a-G-AuNPs and a-DG-AuNPs produces nanoparticle aggregates that after sonication at 45 MHz, yield monodispersed a-G-AuNPs and a-DG-AuNPs of 239 ± 73 nm and 254 ± 64 nm found stable for one week at physiological pH (4–7) in a wide range of temperature. The a-G-AuNPs and a-DG-AuNPs are efficient quenchers of UV–Vis-NIR radiation; both the intrinsic luminescence and the fluorescence of fluorophores, covalently bound to the surface (dansyl group) or in solution (l-tryptophan), are completely quenched. The a-G-AuNPs and a-DG-AuNPs are readily internalized in Hep G2 and murine neuronal cells without affecting cell viability in 24 h. A “*residual*” fluorescence of DG-AuNPs under confocal microscope by irradiation at 488 nm still allows cell imaging. The a-DG-AuNPs induce the formation of micrometric bubbles when excited for 1.26 s at 760 nm with pulsed laser at power of 24 W/cm^2^ because of NIR radiation harvesting of the fluorophore at laser power close to that observed for gold nanorods. The expected fluorescence in the UV–Vis range of the dansyl group under NIR two-photon excitation is efficiently quenched and converted into heat, likewise the direct one-photon UV–Vis excitation at the plasmonic resonance. Thus, the dansyl group acts as an antenna in capturing the multiphoton NIR excitation^40,47^, which is transformed efficiently in a non-radiative thermal decay. Two-photon excitation with pulsed NIR laser is thus a valid alternative to CW excitation in photothermal therapy of cancer cell, preserving the deep tissue penetration of the radiation with a reduced photobleaching. Normal human tissues present a scarce presence of chromophore groups responsive to two-photon NIR excitation. On the basis of these results, it can be envisioned that aggregates of spherical metal nanoparticle functionalized with NIR responsive chromophores are promising candidates for novel devices suitable in theranostics.

## Methods

### Materials

Tetrachloroauric acid trihydrate (Sigma-Aldrich, 99.9%), oxidized glutathione (Sigma-Aldrich, 98% HPLC), reduced glutathione (G; Sigma-Aldrich, 98.0%), sodium hydroxide (Sigma-Aldrich, 98.0%), sodium borohydride (Sigma-Aldrich, 98.0%), sodium chloride (Sigma-Aldrich, 99.8%), dansyl chloride (Sigma-Aldrich, 99.0%), dithiothreitol (TCI, 98.0%), hydrogen peroxide 30% water solution (Carlo Erba), sulfuric acid (Sigma-Aldrich, 99.9%), hydrochloric acid (Sigma-Aldrich, 37%), water (HPLC grade, Sigma-Aldrich), methanol (pure HPLC, Sigma-Aldrich), propidium iodide (PI; Thermo Scientific) were used as purchased without further purifications. Deuterated solvents were purchased from Eurisotop or Sigma-Aldrich. Human HepG2 cell line, derived from a liver hepatocellular carcinoma, were purchased from American Type Culture Collection (Manassas, VA). The cells were incubated with: PBS 1× (*Dulbecco’s phosphate buffered* saline) without calcium e magnesium, at pH of 7.4 containing NaCl (137 mM), KCl (2.7 mM); Na_2_HPO_4_ (10 mM); KH_2_PO_4_ (1,8 mM); Trypsin–EDTA: 500 mg/L trypsin (1:250); 200 mg/L EDTA in PBS; pH 7.4 MEM (Minimum Essential Medium) with l-glutamine Earle’s Minimum Essential Medium (MEM; Biochrom AG); fetal bovine serum (FBS;Thermo Fisher Scientific); non-essential amino acids (Thermo Fisher Scientific); trypsin-ethylenediaminetetraacetic acid (EDTA) (0.025%/0.01% w/v; Biochrom); 1% penicillin–streptomycin-neomycin antibiotic mixture (PSN, Thermo Fisher Scientific, Waltham, MA, USA); poly-l-lysine (0.1 mg/mL; Sigma, St. Louis, MO, USA); neurobasal medium (Thermo), B-27 (Thermo). The gold (III) standard solution (1.000 ± 0.002) g/L in water with HCl (2 wt%) used in inductively coupled plasma-optical emission spectrometry (ICP-OES) analyses was purchased from Carlo Erba and used as received. All reagents used for electron microscopy were purchased from Electron Microscopy Science (Hatfield, PA).

### Instrumentation

Reaction products were characterized by nuclear magnetic resonance (NMR) analysis, performed on Bruker Avance spectrometers (300, 400 and 600 MHz for ^1^H). Powder wide angle x-ray diffraction (WAXD) patterns were obtained in reflection mode with an automatic Bruker D8 powder diffractometer, using the Ni-filtered Cu Kα radiation. The size and zeta potential of gold colloids and nanoparticle aggregates were measured by dynamic light scattering (DLS) performed on a Zetasizer Nano-ZS from Malvern Instruments. The size controlled preparation of a-G-AuNPs and a-DG-AuNPs was assessed after sonication of PBS solution ([Au] = 700 μg/mL) at variance of pH (1.5, 2.4, 4.0, 5.8, 7.0, 11.4 and 12.4) and temperature (25–75 °C). The UV–Vis and fluorescence spectra were recorded on a Varian Cary 50 UV–VIS spectrophotometer and a Varian Cary Eclipse spectrophotometer. The thermogravimetric analyses (TGA) was performed on a TG 209 F1 thermoanalyzer from Netzsch at heating rate of 10 °C/min in oxygen atmosphere. Gold concentration was determined by inductively coupled plasma optical emission spectrometry (ICP-OES) with a Perkin-Elmer Optima 7,000 DV spectrometer. Transmission electron microscopy (TEM) was carried out with a Tecnai Spirit transmission electron microscope from FEI, working at acceleration voltage of 120 kV, equipped with a lanthanum hexaboride thermionic source and a twin objective lens. TEM images were acquired using a Gatan Orius CCD camera from Pleasanton. TEM grids (carbon film supported by 300-meshes copper) were supplied by TedPella (USA). High-resolution confocal microscopy was performed with a Leica TCS-SP2 confocal system equipped with Ar/Kr and He/Ne continuous wave lasers for excitation at 488 nm and 546 nm, respectively. NIR photoablations of Hep G2 cells treated with AuNPs were performed with a Chamaleon (Coherent) or a Mai Tai DeepSee™ eHP (Spectra Physics) pulsed lasers tuned at 760 nm.

### Synthesis of G-AuNPs

Spherical G-AuNPs were synthesized according to a modified literature procedure^23,24^. A 100 mL round bottom flask, equipped with magnetic stir bar, was charged sequentially at room temperature with tetrachloroauric acid (0.333 g, 0.846 mmol), methanol (27.8 mL), water (22.2 mL), reduced glutathione (0.581 g, 1.89 mmol) and sodium hydroxide (0.370 g, 9.25 mmol). The addition of reduced glutathione causes turbidity that disappears after the addition of sodium hydroxide, yielding a clear solution. The solution was transferred at room temperature into a 3 L round bottom flask, diluted with 260 mL of methanol and 760 mL of water, treated rapidly with 15 mL of a freshly prepared aqueous solution of sodium borohydride (0.145 g, 3.83 mmol) under vigorous stirring that causes the formation of a dark brown colloidal suspension. After stirring at room temperature for 48 h, sodium chloride (21.2 g, 0.363 mol) and 700 mL of methanol were added and the suspension transferred into a glass Imhoff cone. G-AuNPs separated from the solution within 48 h and were recovered by removal of the surnatant suspension followed by drying in vacuo. Yield 0.35 g.

### Synthesis of DG-AuNPs

A 100 mL round bottom flask, equipped with magnetic stir bar, was charged sequentially at room temperature with tetrachloroauric acid (0.333 g; 0.846 mmol), methanol (27.8 mL), water (22.2 mL), dansylated reduced glutathione^37,48^ (1.02 g, 1.89 mmol) and sodium hydroxide (0.518 g, 13 mmol). The addition of reduced glutathione causes turbidity that disappears after the addition of sodium hydroxide. The solution was transferred at room temperature into a 3 L round bottom flask, diluted with 260 mL of methanol and 760 mL of water, treated rapidly under vigorous stirring with 15 mL of a freshly prepared aqueous solution of sodium borohydride (0.145 g, 3.83 mmol) that causes the formation of a dark brown colloidal suspension. After stirring for 48 h at room temperature sodium chloride (21.2 g, 0.363 mol) and 700 mL of methanol were added and the suspension transferred into a glass Imhoff cone. DG-AuNPs precipitated within 48 h and were recovered by removal of the surnatant suspension and drying in vacuo. Yield 0.25 g.

### Cell and tissue cultures

Cells were cultured in Eagle's minimum essential medium supplemented with 10% (v/v) fetal bovine serum, 1% non-essential amino acids, 2 mM l-glutamine, 100 U/mL penicillin and 100 mg/mL streptomycin at 37 °C in a humidified atmosphere of 5% CO_2_. The cells were plated onto 20-mm round coverslips at density of 10^5^ cells/well in the same culture medium one day before the experiments. Co-cultures of neurons and astrocytes were obtained from E18 C57/bl6 mice as described in literature^49^. Briefly, after dissection the brain tissue was incubated for 10 min at 37 °C in PBS containing trypsin–EDTA (0.025%/0.01% w/v) and then mechanically dissociated at room temperature with a fire-polished Pasteur pipette. Cell suspension was harvested and centrifuged at 235×*g* for 8 min. Then, the pellet was suspended in 88.8% MEM, 5% FBS, 5% horse serum, 1% glutamine (2 mM), 1% PSN and glucose (25 mM). Cells were plated at a density of 10^5^ cells on 20-mm coverslips and pre-coated with poly(l-lysine) (0.1 mg/mL). 24 h later, the culture medium was replaced with a mixture of 96.5% neurobasal medium, 2% B-27, 0.5% glutamine (2 mM), and 1% PSN. After 72 h, this medium was replaced with a glutamine-free version of the same medium, and the cells were grown for 10 days before carrying-out experiments. Either Hep G2 cells or murine neuronal cultures were incubated with AuNPs (from 70 to 700 μg/mL) in their respective culture media for a variable time period ranging from 1 to 24 h. After this treatment the cells were washed and analysed for: (i) toxicity assay of G-AuNPs and DG-AuNPs to Hep G2; (ii) determination of gold uptake in Hep G2; (iii) investigation on AuNPs internalization in Hep G2 (by means of TEM and confocal microscopy) or murine neuronal cells (by means of confocal microscopy); (iv) assessment of the fluence threshold for the generation of the photothermic effect by G-AuNPs and DG-AuNPs after exposition to pulsed laser tuned at wavelength of 760 nm (in presence of murine neuronal cells); (v) investigation of the cellular mortality of Hep G2 cells after incubation with G-AuNPs, DG-AuNPs, (DG_1_/G_3_)-AuNPs and exposition to pulsed laser tuned at 760 nm under variable incident power. Brain organotypic slice (350 µm-thick) cultures were prepared from hippocampi of P4–P7 rats through a McIllwain tissue chopper and then placed on semiporous membranes (Millipore) for 5–7 days before experiments^29^.

### Toxicity assay of G-AuNPs and DG-AuNPs in Hep G2

Cell viability was evaluated in Hep G2 cells after treatment with a-G-AuNPs and a-DG-AuNPs. 2 mL of sonicated AuNPs suspension (800 μg/mL) were added to each cell plate and incubated for different times. After this treatment, the medium was removed and cells washed with PBS and then incubated with trypsin (0.1% in PBS, final volume 2 mL) for 2 min before adding MEM (1 mL) and pipetting for several times. The cells were transferred into a 15 mL conical tube and centrifuged at 1,200 rpm for 5 min. After removal of the supernatant liquid, the pellet was suspended in MEM (1 mL). 10 µL of this suspension were transferred into a 1.5 mL vial and 10 µL of trypan blue (that evidence in blue dead cells) and 10 µL of PBS were added. The cell counting was carried out with an optical microscope equipped with a Bürker chamber.

### Determination of gold content in AuNPs and gold uptake in the cells

Gold concentration in the synthesized nanoparticles and the gold uptake in Hep G2 cells were determined by ICP-OES. The sample to be analysed was acid digested in a Kjeldahl flask by treatment with concentrated sulfuric acid (2.5 mL, 98 wt%) at 250 °C for 30 min and then with hydrogen peroxide (4.0 mL, 30 wt%) at room temperature. The resulting suspension was heated at 250 °C until to produce a clear solution. Aqua regia (1.5 mL) was added at room temperature and the solution was diluted with an aqueous solution of HCl (10 vol%) to a final volume of 10.0 mL. Calibration was performed using seven standard solutions of Au(III) prepared by progressive dilution of a standard solution (1.000 ± 0.002 g/L in water with 2 wt% of HCl) with water and an aqueous solution of HCl (10 wt%).

### TEM imaging

Specimens of G-AuNPs and DG-AuNPs for TEM imaging were prepared by dispersion of the solid sample in ethanol and deposition of the resulting suspension onto a carbon film supported by 300-meshes copper TEM grids. Specimens for TEM imaging of Hep G2 cells incubated with a-G-AuNPs and a-DG-AuNPs were prepared as follows. After treatment of the cells with AuNPs (700 and 70 µg/mL in a final volume of 1 mL) for 1 h and 2 h, Hep G2 cells were fixed as a monolayer in 2% glutaraldehyde in cacodylate buffer 0.1 mol/L pH 7.4 for 30 min at room temperature, then scraped and pelleted. The cell pellets were further fixed for 24 h at 4 °C and post-fixed for 1 h using a reduced osmium solution prepared by combining 3% potassium ferricyanide with an equal volume of 4% aqueous osmium tetroxide in 0.3 M cacodylate buffer. At the end of the first incubation with heavy metals-based solutions, the cell pellets were washed with bi-distilled water at room temperature and then further incubated for 30 min in 2% osmium tetroxide water solution. After several washings in bi-distilled water the pellets were placed in 1% uranyl acetate water solution and left overnight at 4 °C. Samples were dehydrated by increasing ethanol series (70%, 80%, 90%, 100%, and acetone for 10 min each) and finally embedded in Epon resin. After curing at 60 °C for 48 h thin sections were obtained by cutting the embedded samples using a Leica UC7 ultramicrotome (Leica Microsystems, Vienna, Austria). The samples were finally collected onto 300-meshes copper grids and imaged by TEM.

### Confocal microscopy

AuNPs-treated cells were fixed with para-formaldehyde (4% in PBS, pH = 7.4) for 10 min at room temperature and then permeabilized with Triton X-100 (0.3% in PBS) for 15 min. Cells were subsequently incubated for 30 min with Alexa Fluor 532 Phalloidine (Thermo Scientific) to stain F-actin (cytoskeleton). After excitation at 488 nm with Ar/Kr laser the fluorescence spectrum was collected in the spectral window 500–650 nm. 20 µm thick confocal Z-stacks were recorded at 0.3-µm intervals and AuNPs internalization was assessed by looking at co-localization between green spots and red F-actin stain.

### Assessment of photothermic effect

Photothermic effect was investigated by exciting the AuNPs-treated cells with pulsed lasers tuned at 760 nm (Chamaleon, Coherent, 90 MHz, 140 fs pulse width, maximum average exit power 1.0 W or Mai Tai DeepSee™ eHP, Spectra Physics, 80 MHz, < 70 fs pulse width, maximum average exit power 2.7 W) and at fluence (F) varying from 30 to 539 W/cm^2^ by a filter wheel placed in front of the laser exit and attached, respectively, to a Leica TCS SP2 or a Nikon A1MP confocal systems. During the experiments, cells were maintained in Tyrode’s medium^49^ and formation of heat-induced bubbles was monitored in the aqueous culture medium. The viability of Hep G2 cells, cultured on glass coverslips, or cells in organotypic brain slices and treated for 1 h with AuNPs suspensions (70 μg/mL and 700 μg/mL, respectively), was monitored after incorporation of propidium iodide (PI) and cell irradiation with pulsed laser. PI was dissolved in the culture medium (at dilution 1:100) and then excited at 561 nm with a diode laser. Emitted fluorescence was collected by GaAsP detector and TRITC filter. Hoechst or DAPI (2 µg/mL in the culture medium) were used to label and identify cell nuclei after excitation at 405 nm. Fluorescence signals were recorded at 40× magnification after 1.26 s lasting cell excitation with pulsed laser (Mai-Tai DeepSee™, Spectra-Physics) tuned at 760 nm varying the maximum incident power. Statistical analyses were carried out using the software Sigmaplot 14.0. One-Way ANOVA with Bonferroni post hoc test was used for multiple comparisons. The *p* values were considered significant if < 0.05.

## Supplementary information

Supplementary Information.
