# Supplementary material for: NIR multiphoton ablation of cancer cells, fluorescence quenching and cellular uptake of dansyl-glutathione-coated gold nanoparticles
Source: Sci Rep. 2020 Jul 9;10:11380. doi: 10.1038/s41598-020-68397-1 (PMC7347844; doi:10.1038/s41598-020-68397-1)
Supplement: Supplementary file 1 — Supplementary Information. [file 41598_2020_68397_MOESM1_ESM.docx]

**NIR Multiphoton Ablation of Cancer Cells, Fluorescence Quenching and Cellular Uptake of Dansyl-Glutathione-Coated Gold Nanoparticles**

Antonio Buonerba,*^ab^** Rosita Lapenta,*^b^* Anna Donniacuo,*^b^* Magda Licasale,*^b^* Elena Vezzoli,*^c^* Stefano Milione,*^b^* Carmine Capacchione,*^b^* Mario Felice Tecce,*^d^* Andrea Falqui,*^c^** Roberto Piacentini,*^ef^** Claudio Grassi*^ef^* and Alfonso Grassi*^b^*

*^a^* Sanitary Environmental Engineering Division (SEED), and Consorzio Inter-universitario Previsione e Prevenzione dei Grandi Rischi (Cu.G.Ri.), Department of Civil Engineering, University of Salerno, Via Giovanni Paolo II, 84084 Fisciano (SA), Italy.

*^b^* Past address: Department of Chemistry and Biology *“Adolfo Zambelli”*, University of Salerno, Via Giovanni Paolo II, 84084 Fisciano (SA), Italy.

*^c^* King Abdullah University of Science and Technology (KAUST), Biological and Environmental Sciences and Engineering (BESE) Division, 23955-6900 Thuwal, Saudi Arabia.

*^d^* Department of Pharmacy, University of Salerno, Via Giovanni Paolo II, 84084 Fisciano (SA), Italy.

*^e^* Department of Neuroscience, Università Cattolica del Sacro Cuore, Rome, Italy.

*^f^* Fondazione Policlinico Universitario A. Gemelli IRCCS, Rome, Italy.

*** Correspondence to: A. Buonerba (abuonerba@unisa.it); A. Falqui (andrea.falqui@kaust.edu.sa) and R. Piacentini (roberto.piacentini@unicatt.it).

**Table of Contents**

[**1. Toxicity Assay** S3](#_Toc42794822)

[**Figure S1.** Viability of Hep G2 cells: a) incubated with a-G-AuNPs (800 μg/mL); b) incubated with a-DG-AuNPs (800 μg/mL); c) control experiments in absence of nanoparticles. (Histogram bar green for alive cells and red for dead cells). S3](#_Toc42794823)

[**2. Thermogravimetric Analysis** S4](#_Toc42794824)

[**Figure S2.** TGA thermogram of reduced glutathione. S4](#_Toc42794825)

[**Figure S3.** TGA thermogram of G-AuNPs (48.3 *wt*%_Au_). S4](#_Toc42794826)

[**Figure S4.** TGA thermogram of DG-AuNPs (58.22 *wt*%_Au_). S4](#_Toc42794827)

[**4. Fluorescence Quenching** S5](#_Toc42794828)

[**Figure S5.** Stern-Volmer plot of fluorescence quenching of tryptophan (1.5∙10^-4^ M) with a-G-AuNPs at 22 and 70 °C. In both experiments fluorescence emission was monitored at 358 nm with excitation at wavelength of 287 nm. Stern-Volmer constants (*K_D_*) were 0.179±0.006 L/mg_AuNPs_ at 22°C and 0.195 ± 0.008 L/mg_AuNPs_ at 70°C, while *R^2^* values for linear fittings were 0.996 at 22°C and 0.994 at 70°C. S5](#_Toc42794829)

[**5. Confocal Microscopy** S6](#_Toc42794830)

[**Figure S6.** Representative images by confocal microscope of murine neuronal cells treated with a-DG-AuNPs (0.5 mL; 700 µg/mL) for 1 h: a) fluorescence image (λ_ex_ = 488 nm); b-l) DIC images acquired after irradiations of 1.26 s with pulsed laser tuned at 760 nm with power of 24 W cm^-2^ at intervals of 5 s. S6](#_Toc42794831)

[**Figure S7.** Representative images by confocal microscope of murine neuronal cells treated with a-G-AuNPs (0.5 mL; 700 µg/mL) for 1 h: a) fluorescence image (λ_ex_ = 488 nm); b-l) DIC images acquired after irradiations of 1.26 s, with pulsed laser tuned at 760 nm with power of 24 W cm^-2^, at intervals of 5 s. S7](#_Toc42794832)

# **1. Toxicity Assay**


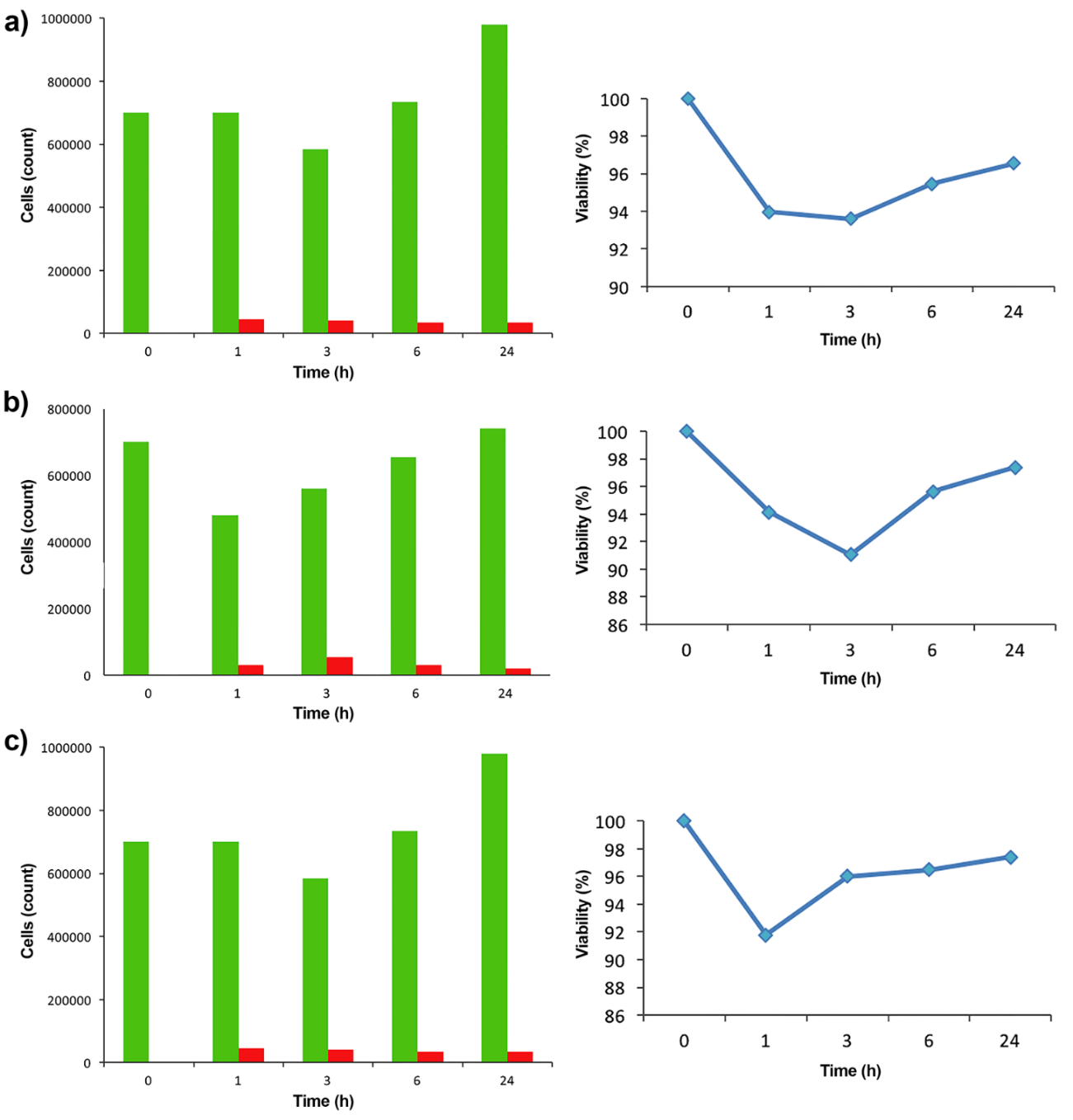


## **Figure S1.** Viability of Hep G2 cells: a) incubated with a-G-AuNPs (800 μg/mL); b) incubated with a-DG-AuNPs (800 μg/mL); c) control experiments in absence of nanoparticles. (Histogram bar green for alive cells and red for dead cells).

# **2. Thermogravimetric Analysis**


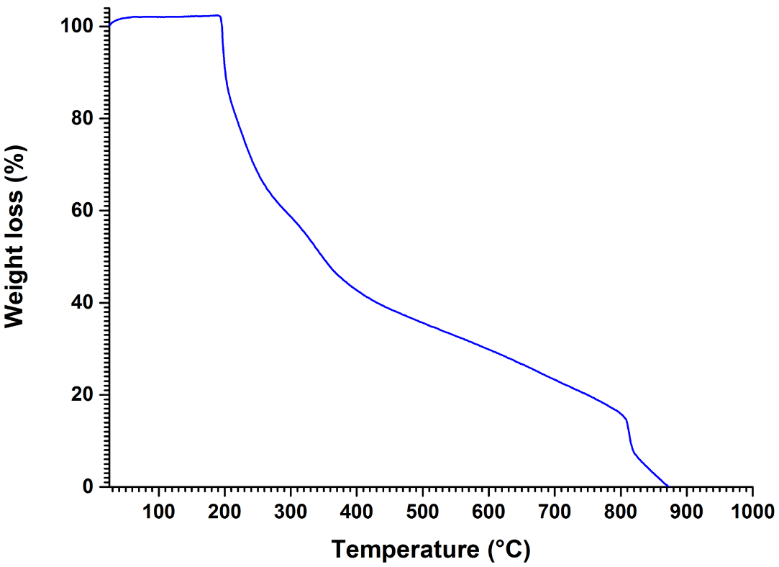


## **Figure S2.** TGA thermogram of reduced glutathione.


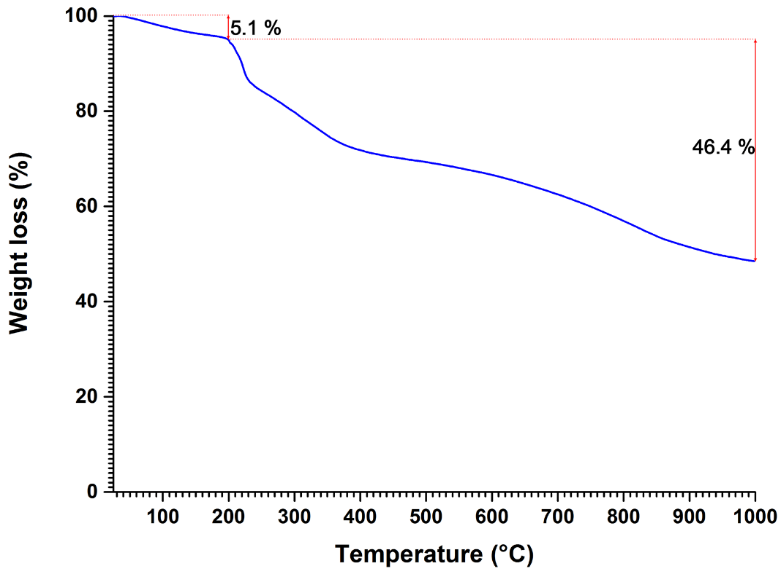


## **Figure S3.** TGA thermogram of G-AuNPs (48.3 *wt*%_Au_).


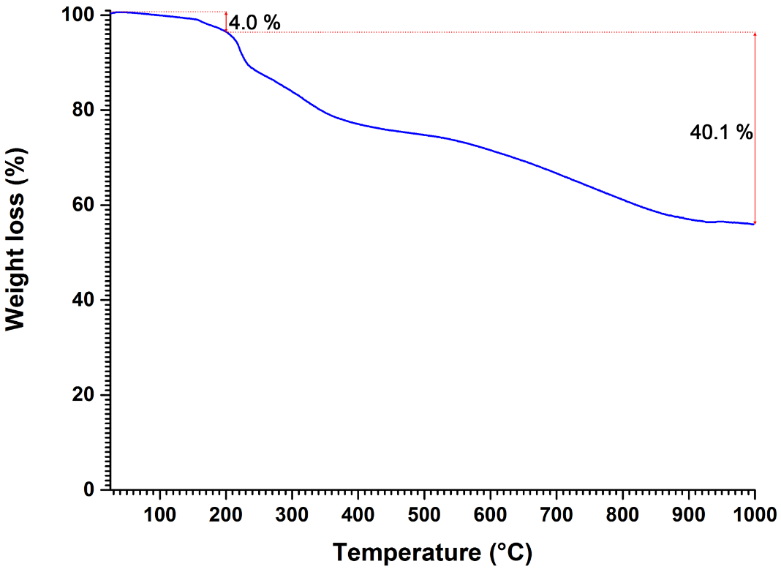


## **Figure S4.** TGA thermogram of DG-AuNPs (58.22 *wt*%_Au_).

# **4. Fluorescence Quenching**





## **Figure S5.** Stern-Volmer plot of fluorescence quenching of tryptophan (1.5∙10^-4^ M) with a-G-AuNPs at 22 and 70 °C. In both experiments fluorescence emission was monitored at 358 nm with excitation at wavelength of 287 nm. Stern-Volmer constants (*K_D_*) were 0.179±0.006 L/mg_AuNPs_ at 22°C and 0.195 ± 0.008 L/mg_AuNPs_ at 70°C, while *R^2^* values for linear fittings were 0.996 at 22°C and 0.994 at 70°C.

# **5. Confocal Microscopy**


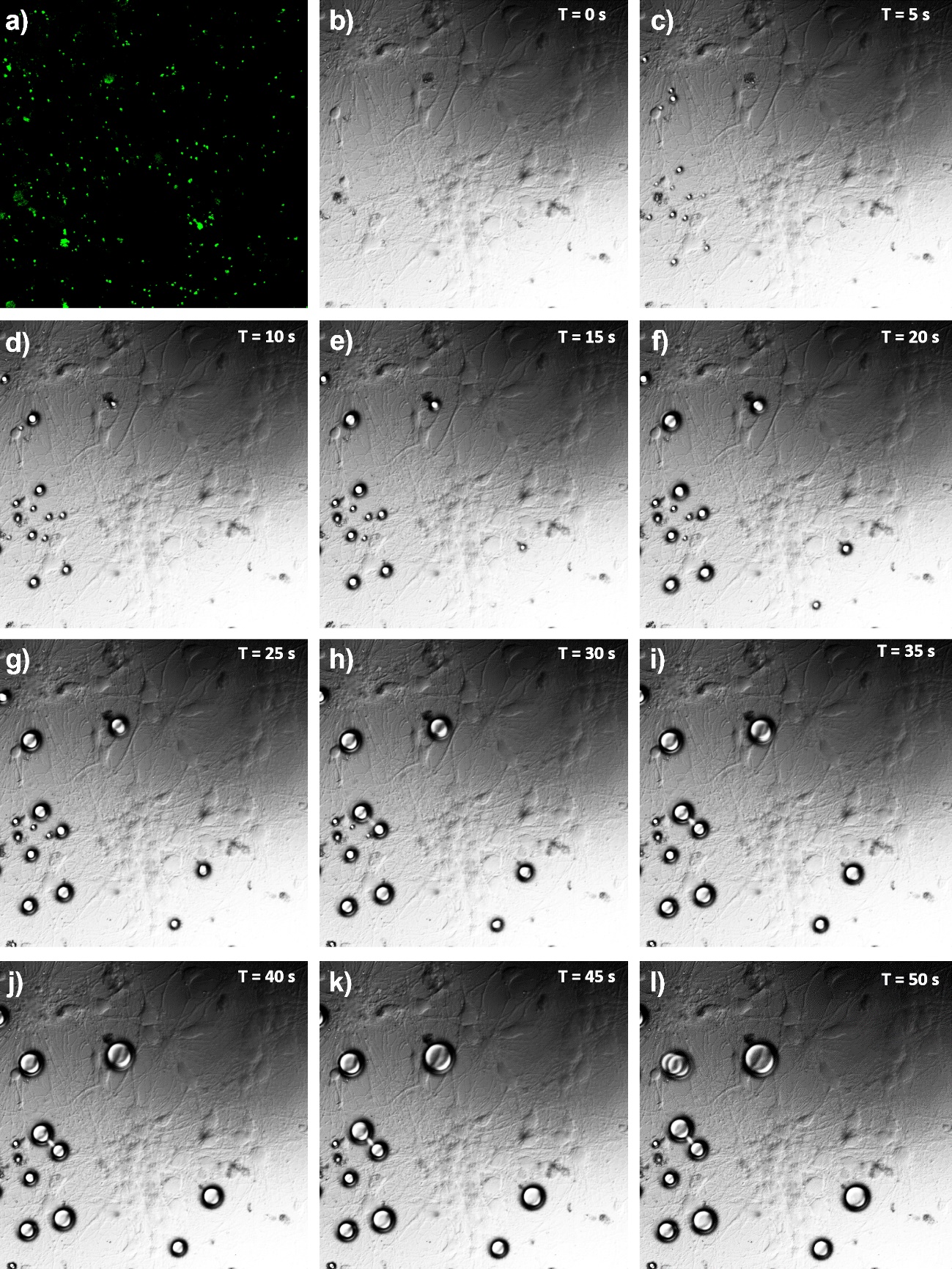


## **Figure S6.** Representative images by confocal microscope of murine neuronal cells treated with a-DG-AuNPs (0.5 mL; 700 µg/mL) for 1 h: a) fluorescence image (λ_ex_ = 488 nm); b-l) DIC images acquired after irradiations of 1.26 s with pulsed laser tuned at 760 nm with power of 24 W cm^-2^ at intervals of 5 s.


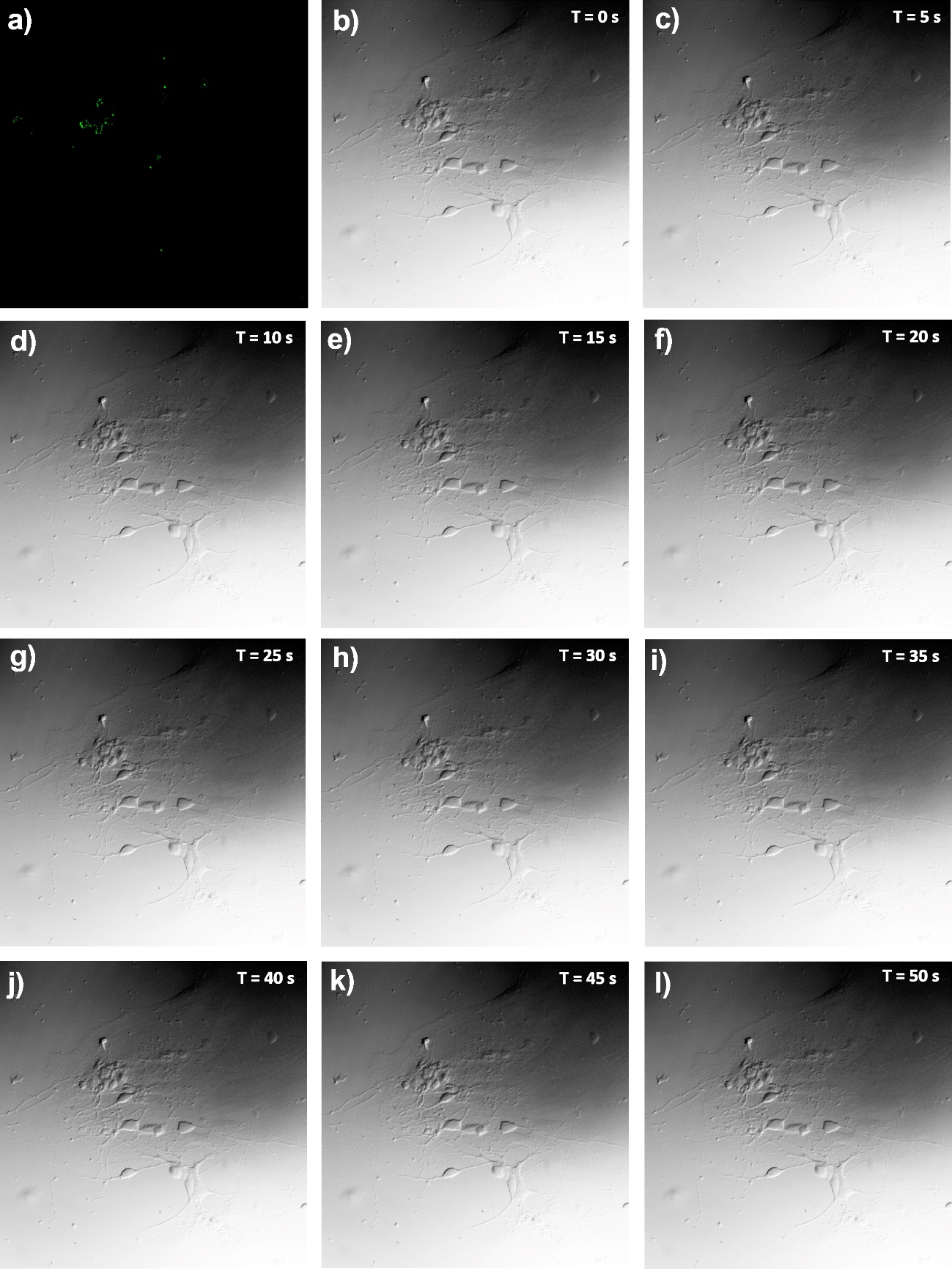


## **Figure S7.** Representative images by confocal microscope of murine neuronal cells treated with a-G-AuNPs (0.5 mL; 700 µg/mL) for 1 h: a) fluorescence image (λ_ex_ = 488 nm); b-l) DIC images acquired after irradiations of 1.26 s, with pulsed laser tuned at 760 nm with power of 24 W cm^-2^, at intervals of 5 s.
